# Survival analysis of laparoscopic surgery and open surgery for hilar cholangiocarcinoma: a retrospective cohort study

**DOI:** 10.1186/s12957-024-03327-3

**Published:** 2024-02-19

**Authors:** Yaolin Yin, Jilin Tao, Yin Xian, Junhao Hu, Yonghe Li, Qiang Li, Yongfu Xiong, Yi He, Kun He, Jingdong Li

**Affiliations:** 1 Department of Hepatobiliary Surgery, Affiliated Hospital of North Sichuan Medical College, Nanchong, 637000 China; 2 Institute of Hepato-Biliary-Pancreatic-Intestinal Disease, North Sichuan Medical College, Nanchong, 637000 China; 3 Department of Hepatobiliary Pancreatic Gastric Surgery, Gaoping District People’s Hospital of Nanchong, Nanchong, 637000 China; 4 Nanchong Psychosomatic Hospital, Nanchong, 637000 China; 5 Clinical Medical College, North Sichuan Medical College, Nanchong, 637000 China

**Keywords:** Laparoscopic surgery, Hilar cholangiocarcinoma, Klatskin tumor, Open surgery, Radical resection; Surgical technique

## Abstract

**Background/purpose:**

This study compared the clinical efficacy and safety of laparoscopic versus open resection for hilar cholangiocarcinoma (HCCA) and analyzed potential prognostic factors.

**Methods:**

The study included patients who underwent HCCA resection at our center from March 2012 to February 2022. Perioperative complications and postoperative prognosis were compared between the laparoscopic surgery (LS) and open surgery (OS) groups.

**Results:**

After screening 313 HCCA patients, 68 patients were eligible for the study in the LS group (*n* = 40) and OS group (*n* = 28). Kaplan-Meier survival curve analysis revealed that overall survival > 2 years and 3-year disease-free survival (DFS) were more common in the LS than OS group, but the rate of 2-year DFS was lower in the LS group than OS group. Cox multivariate regression analysis revealed age (< 65 years), radical resection, and postoperative adjuvant therapy were associated with reduced risk of death (hazard ratio [HR] = 0.380, 95% confidence interval [CI] = 0.150–0.940, *P* = 0.036; *HR* = 0.080, 95% *CI* = 0.010–0.710, *P* = 0.024 and *HR* = 0.380, 95% *CI* = 0.150–0.960, *P* = 0.040), whereas preoperative biliary drainage was an independent factor associated with increased risk of death (*HR* = 2.810, 95% *CI* = 1.130–6.950, *P* = 0.026). Perineuronal invasion was identified as an independent risk factor affecting DFS (*HR* = 5.180, 95% *CI* = 1.170–22.960, *P* = 0.030).

**Conclusions:**

Compared with OS, laparoscopic HCCA resection does not significantly differ in terms of clinical efficacy. Age (**<**65 years), radical resection, and postoperative adjuvant therapy reduce the risk of death, and preoperative biliary drainage increases the risk of death.

**Supplementary Information:**

The online version contains supplementary material available at 10.1186/s12957-024-03327-3.

## Introduction

Hilar cholangiocarcinoma (HCCA), also known as Klatskin’s tumor, is a predominant malignant tumor of the biliary system, accounting for 60–80% of cholangiocarcinomas [1]. Its incidence varies geographically, with the highest in Southeast Asia, and relatively rare occurrences in Europe and the Americas [2]. Typically presenting with painless jaundice, most patients are asymptomatic initially, leading to late-stage diagnoses and poor prognoses with 5-year survival rates of only 20–40% [3–5]. Although a variety of treatments like chemotherapy, radiotherapy, targeted therapy, immunotherapy, and other biological therapies exist, they only prolong the survival for patients with advanced HCCA. To date, radical surgical resection remains the sole curative strategy [6–9].

When it comes to surgical resection, the traditional surgical modality has been open surgery (OS), which, while effective, is often associated with large wounds, extended postoperative hospital stays, and numerous complications. In contrast, laparoscopic surgery (LS) has emerged as a newly developed minimally invasive method, showing promise for better outcomes, though its efficacy and safety are not yet fully established due to limited research. OS is widely performed in general medical centers, whereas radical LS resection of HCCA is predominantly undertaken at institutions with significant expertise in abdominal LS due to the technical challenges posed by the anatomic location of the tumor and the biological characteristics of HCCA [10]. Consequently, only a select group of HCCA patients are suitable candidates for LS. The difficulty associated with laparoscopic HCCA surgery and the uncertainty regarding postoperative outcomes has led to a lack of consensus and ongoing debate concerning the adoption and development of this technique.

In the past decade, our center has treated 313 patients with HCCA. This study aims to fill a gap in the existing literature by comparing the clinical efficacy and safety of LS versus OS for HCCA, and identifying factors that influence postoperative prognosis, thereby contributing to the optimization of treatment strategies for this challenging condition.

## Materials and methods

### Study design and patient selection

**Fig. 1:**
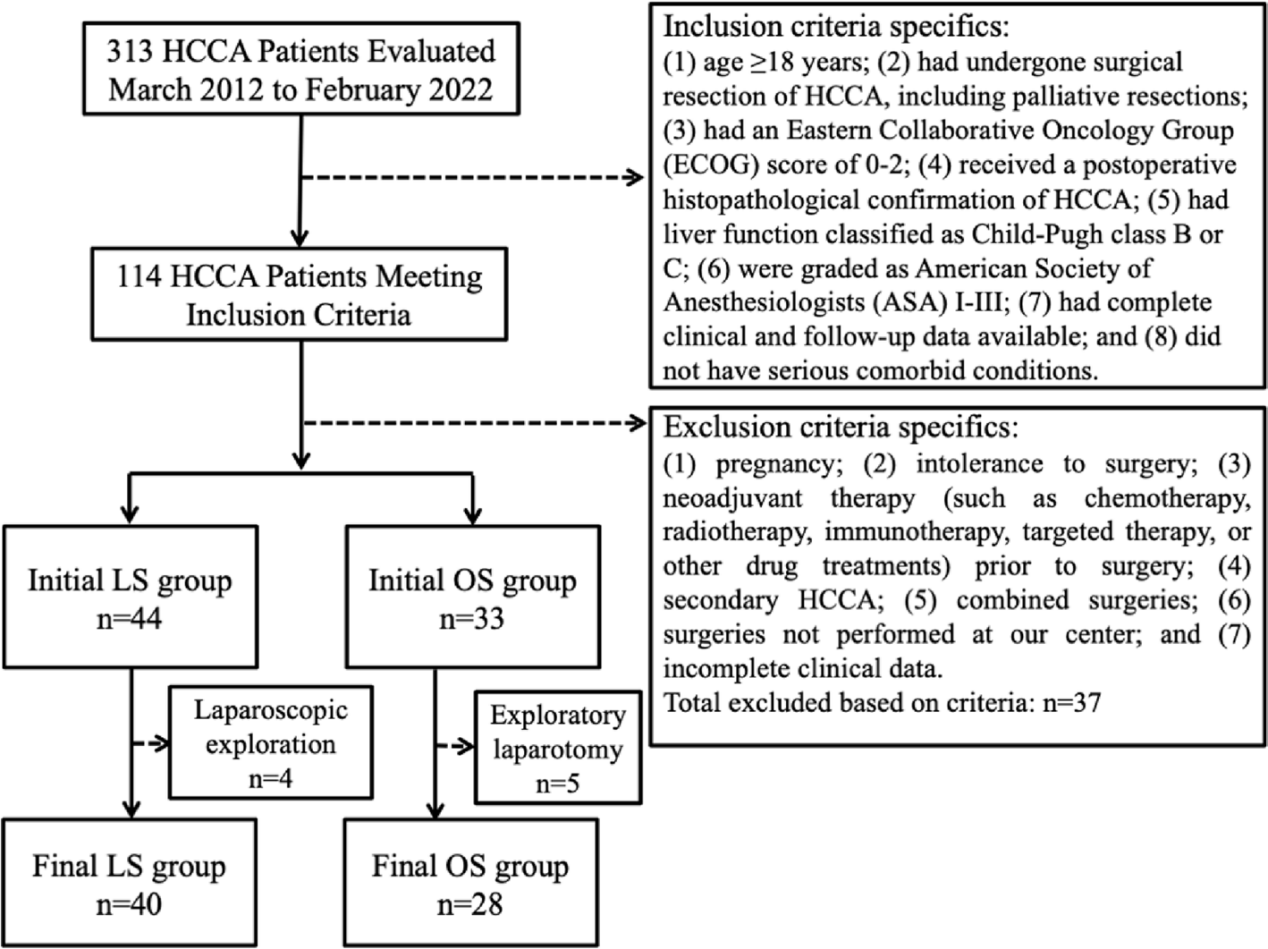
Case inclusion flowchart for detailing the selection process for HCCA patients in LS and OS groups

In this retrospective cohort study, we aimed to compare perioperative complications and survival outcomes between patients undergoing LS and OS for HCCA. Between March 2012 to February 2022, our hospital treated 313 patients with HCCA. The data were stored in a specialized hepatobiliary pancreatic tumor database. Initially, 114 patients met our inclusion criteria, but after applying our exclusion criteria, the final study cohort consisted of 68 patients, divided into the LS (*n* = 40) and OS (*n* = 28) groups (Fig. 1).

### Inclusion and exclusion criteria

Patients were included if they were as follows: (1) age ≥ 18 years; (2) had undergone surgical resection of HCCA, including palliative resections; (3) had an Eastern Collaborative Oncology Group (ECOG) score of 0–2; (4) received a postoperative histopathological confirmation of HCCA; (5) had liver function classified as Child-Pugh class B or C; (6) were graded as American Society of Anesthesiologists (ASA) I–III; (7) had complete clinical and follow-up data available; and (8) did not have serious comorbid conditions affecting the heart, lung, brain, or kidney. Tumor staging was performed using the Bismuth-Corlette classification, with particular attention to type IV tumors and any necessity for vascular reconstruction. This Bismuth-Corlette classification has been internationally recognized for the classification of hilar cholangiocarcinoma. In our cohort, patients typically exhibited elevated bilirubin levels due to the obstructive nature of the tumor, leading to their categorization as Child-Pugh class B or C. As a result, no patients with Child-Pugh class A were included in this study.

Exclusion criteria were as follows: (1) pregnancy; (2) intolerance to surgery; (3) neoadjuvant therapy (such as chemotherapy, radiotherapy, immunotherapy, targeted therapy, or other drug treatments) prior to surgery; (4) secondary HCCA; (5) combined surgeries; (6) surgeries not performed at our center; and (7) incomplete clinical data, such as missing visits, incomplete serological reports, and lack of imaging data.

This study is a retrospective cohort analysis wherein the patients were allocated to either the LS or OS group based on the type of completed surgery they underwent. This differs from prospective studies where patients are randomly assigned to specific surgical interventions. The selection for LS was guided by an exhaustive preoperative evaluation and intraoperative exploration, conducted by our multidisciplinary team. This process was underpinned by the specific oncological and anatomical challenges associated with HCCA. Preoperative imaging, including CT, MRCP, CT angiography, or MR angiography, was crucial in assessing the tumor’s relationship with the hepatic artery and portal vein and determining the presence or extent of invasion. Criteria for choosing LS encompassed patients with Bismuth-Corlette types I and II, select cases of type III, and part of type IV tumors, specifically those without portal vein and hepatic artery invasion. Conversely, contraindications for LS, aside from the general contraindications for OS, included inability to tolerate or establish pneumoperitoneum, extensive abdominal adhesions, significant tumor invasion into the portal vein or hepatic arterial trunk, and the presence of regional portal hypertension in the hepatic hilum which could preclude safe radical resection.

We acknowledge the inherent limitations of retrospective analyses, including the potential for selection bias in allocating patients to surgical groups based on completed procedures. To mitigate this, our multidisciplinary team employed stringent criteria based on preoperative imaging and intraoperative findings to guide the decision-making process. During surgery, if the tumor’s characteristics or patient’s anatomy deviated from our preoperative assessments, necessitating a change in the surgical approach, such cases were meticulously documented. This ensured that our comparative analysis between LS and OS groups was as robust as possible within the constraints of a retrospective design.

### Ethics and consent

As a retrospective study, informed consent for participation was waived by the Medical Ethics Committee of our hospital, in compliance with ethical standards. The study adhered to the Declaration of Helsinki principles. The relevant data for this study were extracted from patient medical records.

### Preoperative preparation

**Fig. 2:**
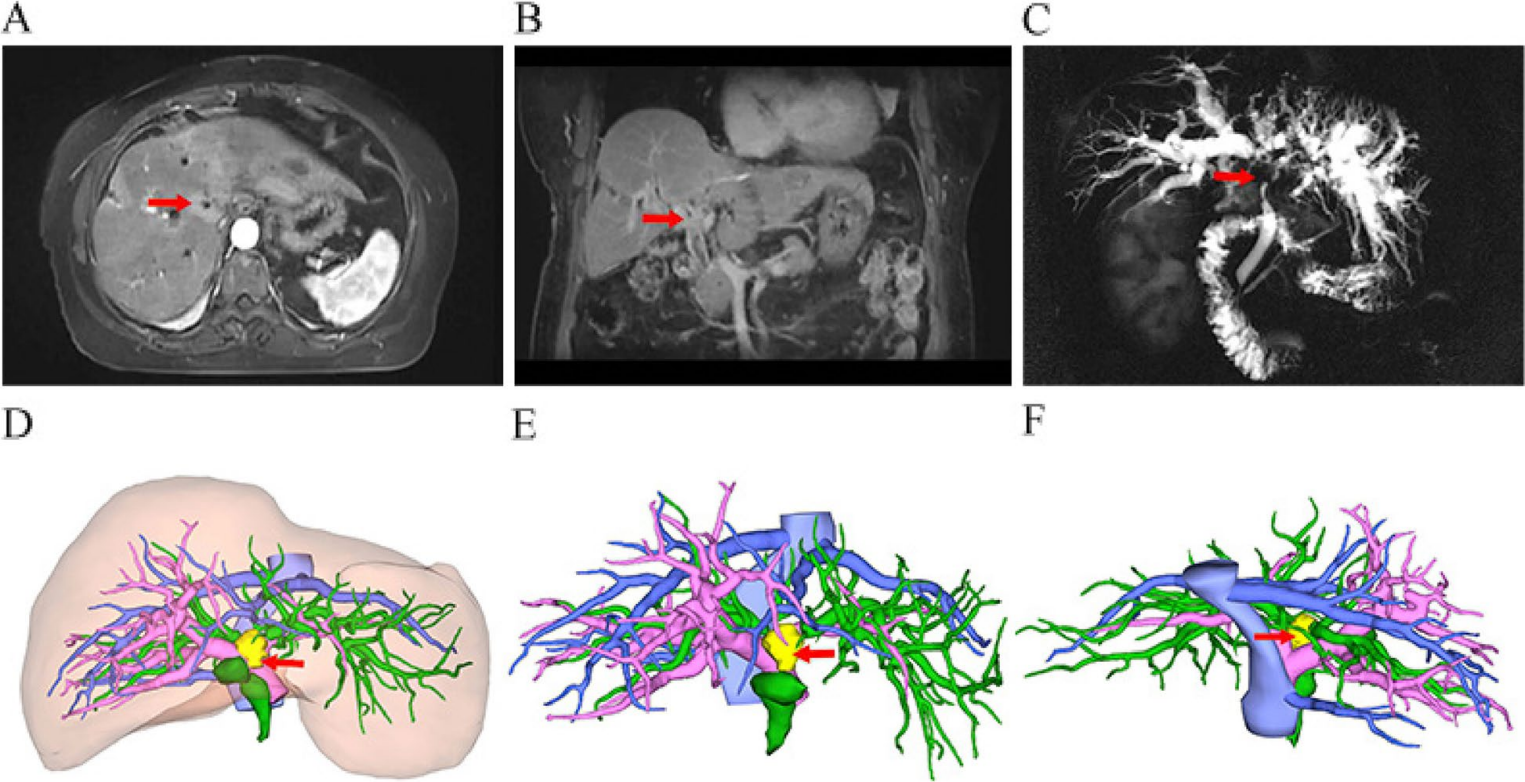
**A** Location of the tumor on the MRI cross-section. **B** Coronal MRI showing the shape of the tumor. **C** MRCP showing the intrahepatic and extrahepatic biliary systems. **D** Three-dimensional reconstruction showing the relationship between the liver and the biliary system, blood vessels, and tumor. **E** Frontal three-dimensional reconstruction showing the relationship between the biliary system, blood vessels, and tumor. **F** Three-dimensional reconstruction of the relationship between the biliary system, blood vessels, and tumor, as shown in lateral view

All patients underwent a comprehensive preoperative assessment to assess the characteristics and staging of the tumor. This included contrast-enhanced computed tomography (CT) or magnetic resonance imaging (MRI), as well as magnetic resonance cholangiopancreatography (MRCP) when indicated. Three-dimensional reconstruction was particularly utilized for those with Bismuth-Corlette type IV HCCA to facilitate detailed preoperative planning (Fig. 2). For patients presenting with obstructive jaundice and serum bilirubin levels exceeding 200 μmol/L, or those suffering from cholangitis or at high nutritional risk, percutaneous transhepatic biliary drainage was the preferred method to alleviate jaundice and optimize their condition before surgery. Informed consent for the surgical procedure was obtained from each patient or their family after a thorough discussion of the risks, benefits, and alternatives to the proposed surgical intervention.

The Bismuth-Corlette classification was initially estimated through imaging studies; however, it was acknowledged that the definitive classification often necessitates intraoperative assessment due to potential deviations from preoperative imaging predictions. A review of our center’s historical data has indicated a trend towards increased utilization of laparoscopic techniques for HCCA over recent years, which is consistent with the increased incidence of higher Bismuth type tumors in our study cohort. Importantly, the majority of patients were diagnosed at an advanced stage, underscoring the need for meticulous preoperative preparation and evaluation.

### Surgical approach

#### Laparoscopic surgery

Before detailing the technical steps of the laparoscopic approach, it is crucial to outline the indications that led to the selection of LS for treating patients with HCCA. Patients were considered eligible for LS based on a combination of factors including, but not limited to, tumor size and location, the absence of extensive vascular involvement, patient’s physiological status, technical feasibility, and the surgeon’s expertise. The multidisciplinary team, including hepatobiliary surgeons, radiologists, and oncologists, evaluated each case to determine the appropriateness of LS. The decision also took into account the patient’s preference and understanding of the potential risks and benefits after thorough preoperative counseling. These indications align with the intention of providing a minimally invasive approach while ensuring patient safety and the best possible oncological outcomes.

**Fig. 3:**
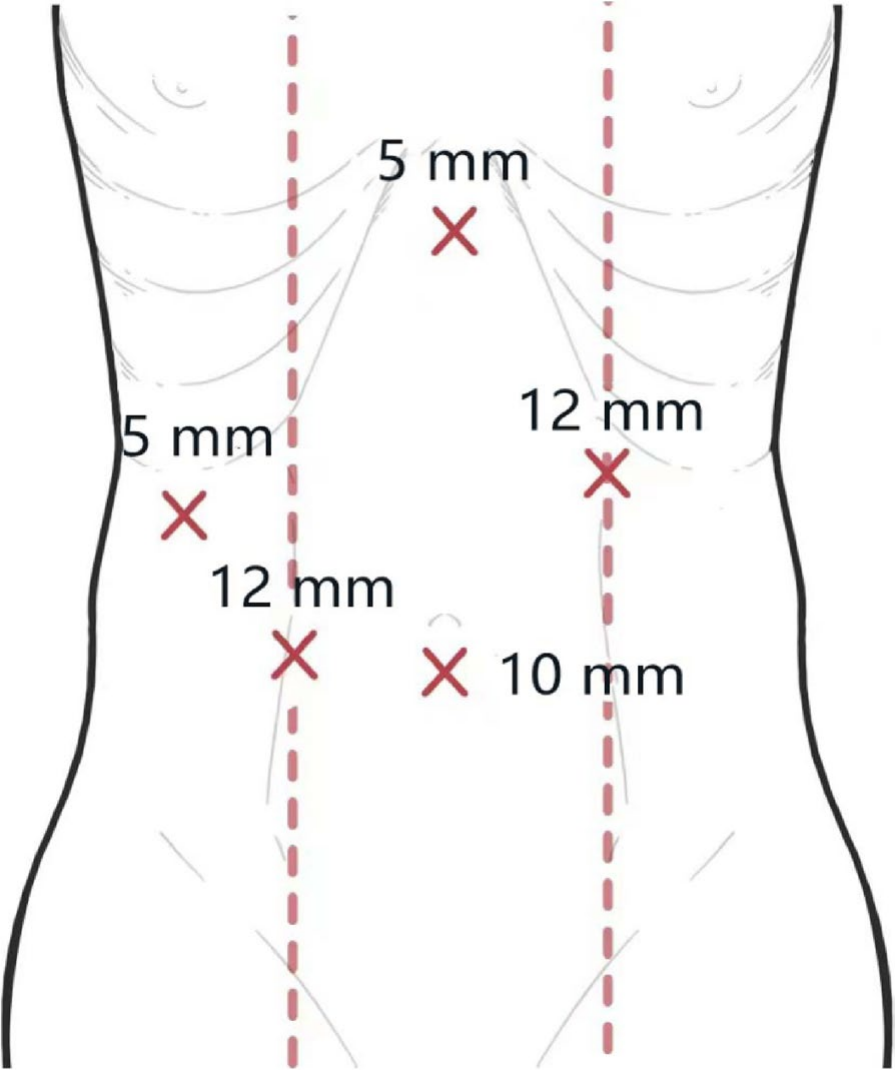
Distribution of trocar locations in Bismuth-Corlette type IV HCCA surgery

**Fig. 4:**
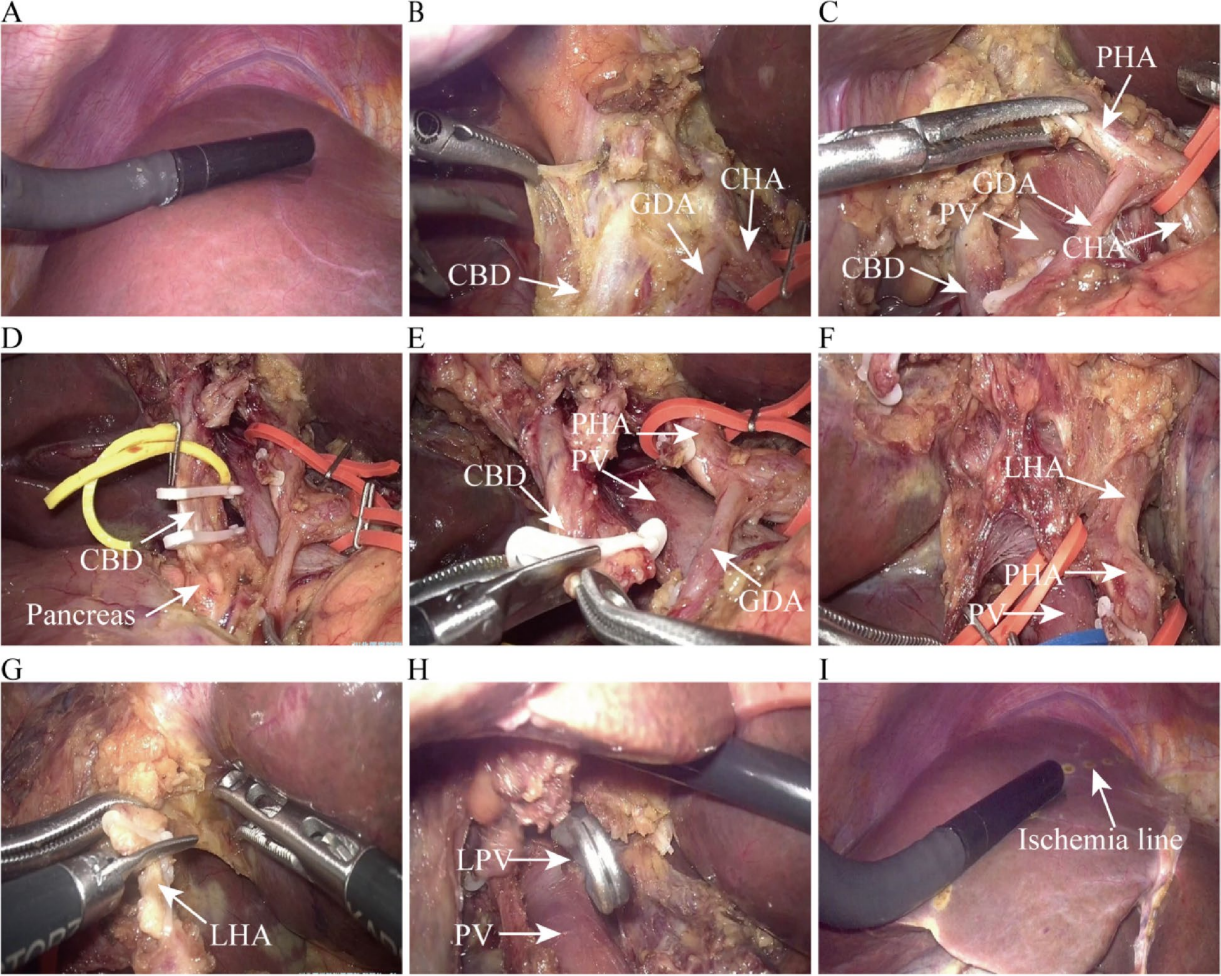
**A** Intraoperative ultrasound comprehensive exploration to evaluate tumors. **B** Dissection and separation of the common bile duct (CBD), common hepatic artery (CHA), and gastroduodenal artery (GDA). **C** Freeing and skeletalizing of the CBD, CHA, and GDA and simultaneous dissection of lymph nodes in groups 8, 12, and 13. **D** Clamping of the CBD with a Hom-Loc clamp from the upper edge of the pancreas, followed by severing. **E** Frozen pathological examination of the distal resection margin of the CBD. **F** Dissection and separation of the right hepatic artery, left hepatic artery (LHA), and portal vein (PV). **G** Double ligation and transection of the LHA. **H** Temporary occlusion of the left portal vein (LPV) with a vascular clip. **I** Observation of change in liver color combined with intraoperative ultrasonography to determine the left hepatic ischemia line. *PHA*, proper hepatic artery

**Fig. 5:**
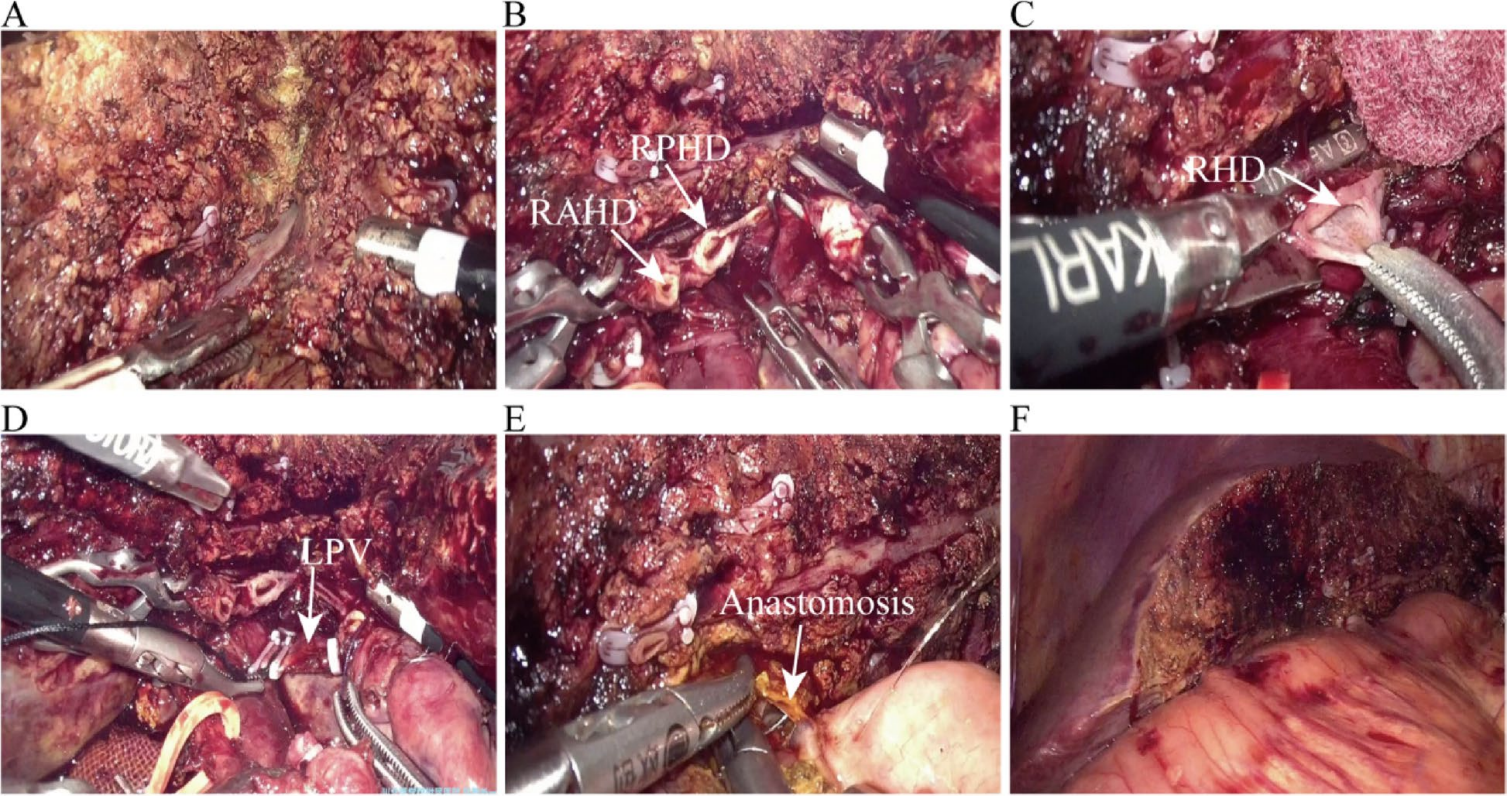
**A** Splitting of the liver and use of a Hom-Loc clip to close the thicker blood vessels. **B** Dissection and transection of the right hepatic duct (RHD). **C** Frozen pathological examination of the proximal bile duct margins. **D** The left portal vein is double-ligated and disengaged. **E** Hepatic duct-jejunal Roux en-Y anastomosis is performed after bile duct molding. **F** Sufficient hemostasis on the cut surface of the liver after completion of biliary anastomosis

Upon determining eligibility for LS and obtaining informed consent, patients were anesthetized and positioned supine with legs apart. The operating area was disinfected and pneumoperitoneum established. The surgical team’s arrangement was consistent, with the senior surgeon to the patient’s right, the first assistant to the left, and the camera assistant at the patient’s feet. Trocar placement followed a conventional five-port approach for Bismuth-Corlette type IV HCCA as depicted in Figs. 3,  4 and 5 outline the critical steps of the LS, focusing on the radical resection of HCCA, starting from intraoperative assessment to the completion of the anastomosis.

### Open surgery

The open surgical approach commenced with the creation of a reversed “L”-shaped incision under the right costal margin, extending approximately 25 cm in length. This incision allowed for systematic exploration of the abdominal cavity, proceeding layer by layer. The remaining surgical steps were the same as for laparoscopic surgery.

### Variables

In order to analyze the factors that affect the prognosis of surgery, this study collected general patient data, laboratory findings, perioperative parameters, imaging, and pathological examination results. General data included gender, age, body mass index (BMI), ECOG score, concomitant diseases, and ASA and Child-Pugh classifications. Laboratory test result data collected included the following: preoperative alanine aminotransferase (ALT) level, aspartate aminotransferase (AST) level, alkaline phosphatase (ALP) level, glutamyl transpeptidase (GGT) level, direct bilirubin (DBIL) level, total bilirubin (TBIL) level, albumin (ALB) level, preoperative coagulation function (prothrombin time [PT], prothrombin international normalized ratio [INR], plasma fibrinogen concentration [FIB], and activated partial thromboplastin time [APTT]), carcinoembryonic antigen (CEA), carbohydrate antigen CA19-9 (CA-199), alpha-fetoprotein (AFP), and liver function 1-week post-surgery (ALT, AST, ALP, GGT, ALB, TBIL, and DBIL). Perioperative parameters included the following: preoperative nutritional support, preoperative biliary drainage, intraoperative blood loss, intraoperative blood transfusion, Bismuth classification, vascular invasion, liver resection, intraoperative presence or absence of hilar blockade, surgical margin status (R0, R1, or R2), operation time, and postoperative complications (liver failure, bile leakage, acute respiratory distress syndrome, abdominal infection, intra-abdominal hemorrhage, and disseminated intravascular coagulation), with or without postoperative blood transfusion, postoperative hospital stay time, and total hospitalization expenses (from admission to discharge). Imaging and pathological findings included the following: histopathological report, abdominal enhanced CT, abdominal ultrasound (US), MRI, and MRCP.

### Endpoints and assessments

Overall survival was defined as the period from the date the patient underwent surgical resection of HCCA to the end of follow-up or date of death. Disease-free survival (DFS) was defined as the time from the date of surgical resection of HCCA to the date of diagnosis of tumor recurrence or the end of follow-up or date of death. In addition, patients were followed up by telephone and outpatient services. According to Clavien-Dindo classification, postoperative complications were classified as grades I, II, III, or IV [11, 12]. Short-term outcomes were evaluated based on postoperative complication rate and mortality within 90 days. Long-term outcome was tumor recurrence or death after 90 days.

### Statistical analysis

Continuous variables are expressed as mean ± standard deviation (SD) or median (range), and the significance of differences between groups was assessed using the Student’s *t*-test or Mann-Whitney *U*-test, respectively. Categorical variables are expressed as frequencies and percentages, and differences between groups were analyzed using Fisher’s exact test or the *χ*^2^ test. Survival was analyzed using the Kaplan-Meier method, with differences evaluated using the log-rank test.

In our study, addressing the crucial aspect of sample size adequacy was paramount, particularly given the unique challenges in accruing large cohorts for HCCA surgery. We performed a rigorous power analysis using PASS software, specifically focused on the median survival outcomes shown from our survival time curves. This analysis yielded a power value of 0.76. We acknowledge that this figure is slightly below the standard threshold of 0.8. However, considering the specific context of HCCA surgery — a field where patient eligibility for either LS or OS is inherently limited due to the complex nature of the disease and technical demands of the procedures — this power value represents a reasonable level of statistical robustness. It indicates our study’s ability to detect significant differences in survival outcomes between the LS and OS groups, despite the challenges in patient recruitment. Our study cohort, encompassing 68 patients from a single center over a decade, represents one of the larger cohorts in this specialized field, as outlined in our “Introduction” and “Discussion”. This cohort size, while smaller than those in more prevalent conditions, is substantial given the rarity and surgical complexity of HCCA.

Univariate and multivariate analyses of overall survival and DFS were performed using the Cox proportional hazard model (variables with a combination of clinical expertise and *P* < 0.20 in the univariate Cox proportional hazards analysis were incorporated into the multivariate Cox proportional hazards analysis). Three-dimensional reconstruction used SYNAPSE 3D (Fujifilm Medical Co., Ltd., Tokyo, Japan). All statistical analyses were performed using R Studio software (version 1.3.1093) and IBM SPSS software (version 23.0). *P* < 0.05 indicated a statistically significant difference.

## Results

### Patient demographic and baseline characteristics

The demographic and baseline characteristics of the two groups are shown in Table 1. ASA score, Child-Pugh classification, and GGT level differed significantly between the LS and OS groups (*P* < 0.05). However, no significant differences were observed in the remaining baseline characteristics (*P* > 0.05).

**Table 1:** Demographic and baseline characteristics of patients in the LS and OS groups

Characteristic	LS group (*n* = 40)	OS group (*n* = 28)	*p*-value
Gender, *n* (%)			
Male	22 (55.0)	21 (75.0)	0.127
Female	18 (45.0)	7 (25.0)	
Age, years, median (IQR)	63 (53.5–68)	64.5 (57.0–69.75)	0.403
BMI, kg/m^2^	22.5 (20.76–23.60)	21.9 (19.76–24.75)	0.596
ECOG-PS, *n* (%)			
0	9 (22.5)	8 (28.57)	0.393
1	25 (62.5)	13 (46.43)	
2	6 (15.0)	7 (25.0)	
ASA score, *n* (%)			
I	13 (32.5)	1 (3.57)	0.002*
II	19 (47.5)	12 (42.86)	
III	8 (20.0)	15 (53.57)	
Child-Pugh classification			
B	32 (80.0)	15 (53.57)	0.032*
C	8 (20.0)	13 (46.43)	
Comorbidity, *n* (%)			
Diabetes	2 (5.0)	3 (10.7)	0.396
Hypertension	8 (20.0)	2 (7.14)	0.179
Cardiac insufficiency	3 (7.5)	2 (7.14)	> 0.999
Pulmonary insufficiency	1 (2.5)	2 (7.14)	0.564
Cerebrovascular disease	0	1 (3.57)	0.412
Viral hepatitis	5 (12.5)	1 (3.57)	0.389
Cirrhosis	3 (7.50)	2 (7.14)	> 0.999
Gallstones	5 (12.5)	3 (10.71)	> 0.999
Cholecystitis	3 (7.50)	7 (25.0)	0.079
Previous abdominal surgery, *n* (%)	6 (15.0)	5 (17.86)	0.751
Preoperative biliary drainage, *n* (%)			
PTCD	22 (55.0)	12 (42.86)	0.460
ERCP	0(0)	1 (3.57)	0.412
Preoperative liver function			
ALT, U/L	64.5 (35.75–127.5)	83.7 (50–134.75)	0.419
AST, U/L	60 (41–101.5)	65.7 (45.85–107.25)	0.414
GGT, U/L	205.85 (114.5–652)	548.1 (307.25–854.5)	0.006*
DBIL, μmol/L	91.3 ± 68.34	97.39 ± 61.12	0.702
TBIL, μmol/L	143.1 ± 100.39	148.2 ± 83.37	0.826
ALB, g/L	38.95 (34.725–42.2)	38.65 (34.0–41.0)	0.529
ALP, U/L	279 (212–597.75)	416.8 (232.8–637.25)	0.124
Preoperative coagulation function			
APTT, s	34.52 ± 4.89	35.46 ± 5.33	0.453
FIB, g/L	4.22 (3.65–4.89)	4.56 (3.62–5.53)	0.246
INR	0.97 (0.91–1.0325)	0.935 (0.9025–1.015)	0.685
PT, s	12.55 (11.625–13.1)	12.5 (11.7–13.5)	0.774
Preoperative tumor markers			
CA-199, U/mL	270.8 (36.10–2581.14)	330.8 (76.46–1385.5)	0.975
CEA, μg/L	2.235 (0.925–5.808)	2.140 (1.013–4.488)	0.627
AFP, μg/L	2.55 (0.90–6.30)	2.16 (1.62–3.865)	0.831

*LS*, laparoscopic surgery; *OS*, open surgery; *IQR*, interquartile range; *BMI*, body mass index; *ECOG-PS*, Eastern Cooperative Oncology Group performance status; *ASA*, American Society of Anesthesiologists; *PTCD*, percutaneous transhepatic cholangial drainage; *ERCP*, endoscopic retrograde cholangiopancreatography; *ALT*, alanine aminotransferase; *AST*, aspartate aminotransferase; *GGT*, gamma-glutamyl transpeptidase; *DBIL*, direct bilirubin; *TBIL*, total bilirubin; *ALB*, albumin; *ALP*, alkaline phosphatase; *APTT*, activated partial thromboplastin time; *FIB*, fibrinogen; *INR*, international normalized ratio; *PT*, prothrombin time; *CA-199*, cancer antigen 19-9; *CEA*, carcinoembryonic antigen; *AFP*, alpha-fetoprotein. “*”Indicates a statistically significant difference (*P* < 0.05)

### Intraoperative technical parameters and surgical decision-making in LS and OS for HCCA

Our retrospective examination revealed distinct intraoperative differences between LS and OS in treating hilar cholangiocarcinoma. Notably, the surgical approach was significantly associated with the extent of hepatectomy, particularly in the frequency of segment I resection and the presence of gallbladder swelling, as well as differences in Bismuth-Corlette classification. Specifically, segment I resection was more commonly executed in the LS group (62.5%) compared to the OS group (25.0%, *P* = 0.003), indicating a predilection for LS in complex liver resections.

Although 61 patients underwent radical resection, the distribution between the LS (*n* = 34) and OS (*n* = 27) groups did not significantly differ. The LS approach was favored for both left hemihepatectomy plus caudate lobectomy and right hemihepatectomy plus caudate lobectomy, highlighting the technical versatility of LS. Despite the LS group initiating with 40 patients, 9 required conversion to OS due to unforeseen intraoperative complications, such as significant bleeding, vascular invasion by the tumor, or anatomical constraints, leading to a conversion rate of 22.5%. These conversions are reflective of the challenging nature of LS and its evolving role in complex hepatic procedures.

Other intraoperative measures, including operation duration, blood loss, and transfusion requirements, showed no statistical disparity, underscoring a comparable level of operative challenge between the two modalities. Additionally, the absence of significant differences in hepatic duct plasty, Roux-en-Y reconstruction, surgical margins, and vascular reconstruction, among other parameters, suggests a uniformity in operative standards and practices irrespective of the surgical approach chosen.

In conclusion, our findings show the surgical intricacies and strategic decisions inherent in LS and OS for hilar cholangiocarcinoma, with the LS group demonstrating a tendency for more extensive hepatic resections. This reflects the detailed surgical considerations and highlights the potential of LS in managing complex liver surgeries.

### Short-term postoperative outcomes and pathology

Our comparative analysis of short-term postoperative outcomes (Table 2) revealed that patients undergoing LS experienced quicker postoperative ambulation and extubation, as well as shorter hospital stays compared to the OS group, underscoring potential advantages in recovery (*P* < 0.05 for all). Notably, liver function tests performed approximately 1-week post-surgery showed no significant differences between groups, indicating comparable organ function recovery post-intervention.

**Table 2:** Short-term postoperative outcomes and pathology results

Variable	LS group (*n* = 40)	OS group (*n* = 28)	*p*-value
Postoperative liver function, *n* (%)			
ALT, U/L	64 (44.5–109.65)	60.05 (33.5–92.5)	0.448
AST, U/L	50.5 (33.525–76.375)	45 (34.5–54.8)	0.389
GGT, U/L	128.5 (61.5–261.5)	122.45 (86.025–198.15)	0.953
DBIL, μmol/L	49.55 (22.15–120.15)	42.45 (24.125–83.3)	0.614
TBIL, μmol/L	86.15 (44.15–187.325)	65.25 (48.35–112.05)	0.351
ALP, U/L	189.15 (140.25–271.25)	207 (118.2–267.225)	0.933
ALB, g/L	34.35 (32.025–38.3)	33.7 (29.55–38.85)	0.426
Postoperative transfusion, *n* (%)			
Plasma, mL	9 (22.5)	11 (39.29)	0.135
Red cells, U	6 (15.0)	6 (21.43)	0.494
ICU admission, *n* (%)	8 (20.0)	4 (14.29)	0.748
Postoperative fasting, days	6.28 ± 3.0	7.22 ± 2.74	0.190
Postoperative ambulation, days	5.43 ± 2.43	7.15 ± 2.16	0.004*
Postoperative extubation, days	12.47 ± 6.54	17 ± 7.30	0.009*
Postoperative hospital stay time, days	17.0 (14–21.75)	19.0 (17.25–29.75)	0.027*
Total hospitalization expenses, RMB	100,218.43 ± 29,216.81	88,572.59 ± 30,971.16	0.119
Pathological differentiation types, *n* (%)			
Highly differentiated	12 (30.0)	11 (39.3)	0.539
Moderately differentiated	20 (50.0)	14 (50.0)	
Poorly differentiated	8 (20.0)	3 (10.7)	
Perineuronal invasion, *n* (%)	7 (17.5)	11 (39.29)	0.055
Intravascular tumor thrombus, *n* (%)	9 (22.5)	5 (17.86)	0.765
Lymph node metastasis, *n* (%)	9 (22.5)	11 (39.29)	0.179
Postoperative complications, *n* (%)			
Yes	28 (70.0)	21 (75.0)	0.786
No	12 (30.0)	7 (25.0)	
Clavien-Dindo grading, *n* (%)			
I/II			
Hepatic failure	1 (2.50)	4 (14.29)	0.151
ARDS	1 (2.50)	0	> 0.999
DIC	1 (2.50)	1 (3.57)	> 0.999
Bile leakage	2 (5.00)	3 (10.71)	0.396
Lymphatic leak	1 (2.50)	0	> 0.999
Abdominal infection	5 (12.50)	5 (17.86)	0.730
Pleural effusion	12 (30.0)	6 (21.43)	0.578
Pulmonary infection	6 (15.0)	5 (17.86)	0.751
Incision infection	1 (2.50)	1 (3.57)	> 0.999
Gastroparesis	3 (7.50)	1 (3.57)	0.638
Gastrointestinal bleeding	0	1 (3.57)	0.412
Renal failure	0	1 (3.57)	0.412
III/IV			
ARDS	1 (2.50)	1 (3.57)	> 0.999
DIC	2 (5.00)	1 (3.57)	> 0.999
Intra-abdominal hemorrhage	1 (2.50)	0	> 0.999
Gastrointestinal bleeding	2 (5.00)	2 (7.14)	> 0.999
Pleural effusion	6 (15.0)	7 (25.0)	0.357
Pulmonary infection	2 (5.00)	3 (10.71)	0.396
Severe drug eruption	1 (2.50)	0	> 0.999
Biliary fistula	0	1 (3.57)	0.412
Abdominal infection	0	2 (7.14)	0.166
90-day death, *n* (%)	7 (17.5)	2 (7.14)	0.289

*LS*, laparoscopic surgery; *OS*, open surgery; *ALT*, alanine aminotransferase; *AST*, aspartate aminotransferase; *GGT*, gamma-glutamyl transpeptidase; *DBIL*, direct bilirubin; *TBIL*, total bilirubin; *ALP*, alkaline phosphatase; *ALB*, albumin; *ICU*, intensive care unit; *RMB*, renminbi; *ARDS*, acute respiratory distress syndrome; *DIC*, disseminated intravascular coagulation. “*”Indicates a statistically significant difference (*P* < 0.05)

Plasma and red blood cell transfusions post-surgery were received comparably between groups, reflecting similar postoperative management needs (*P* > 0.05). The ICU occupancy rates post-surgery were higher in the LS group, though not statistically significant, suggesting a trend that may require further exploration. Similarly, while the LS group had non-significantly higher total hospitalization expenses, it warrants attention to the cost implications of surgical choices.

Pathologically, no significant differences were identified in tumor differentiation, lymph node metastasis, perineuronal invasion, or intravascular tumor thrombus presence between groups, indicating uniformity in disease characteristics across surgical approaches (*P* > 0.05). Moreover, the rate of postoperative complications was comparable between LS and OS, which speaks to the overall procedural safety. However, we observed a nonsignificant trend towards higher 90-day postoperative mortality in the LS group, a finding that calls for ongoing scrutiny into perioperative care (*P* > 0.05).

### Long-term postoperative outcomes

Evaluating the long-term survival, the LS group demonstrated 2-year and 3-year overall survival rates of 42.0% and 38.2%, respectively, with disease-free survival (DFS) rates of 48.8% and 43.4%. For the OS group, these rates were 29.7% and 19.8% for overall survival and 52.2% and 41.8% for DFS at the 2-year and 3-year marks, respectively. These differences did not reach statistical significance, indicating comparable long-term efficacy between the surgical approaches (*P* > 0.05).

The median follow-up time was slightly lower in the LS group compared to the OS group, with IQR providing a measure of dispersion and a more robust representation of the follow-up period. Despite these variations, the maximum follow-up times were extensive, reaching over 77 months for LS and 97 months for OS, suggesting a comprehensive observational span for both groups.

The administration of adjuvant therapy post-surgery was comparable between the two groups, as was the pattern of tumor recurrence, with no significant difference in the occurrence of local relapse or distant metastases between LS and OS groups (*P* > 0.05). However, a significant difference emerged in the mortality rate within 90-day post-surgery, with the OS group exhibiting a higher rate, a finding that may have implications for surgical decision-making (55.0% LS vs. 82.14% OS, *P* = 0.036).

**Fig. 6:**
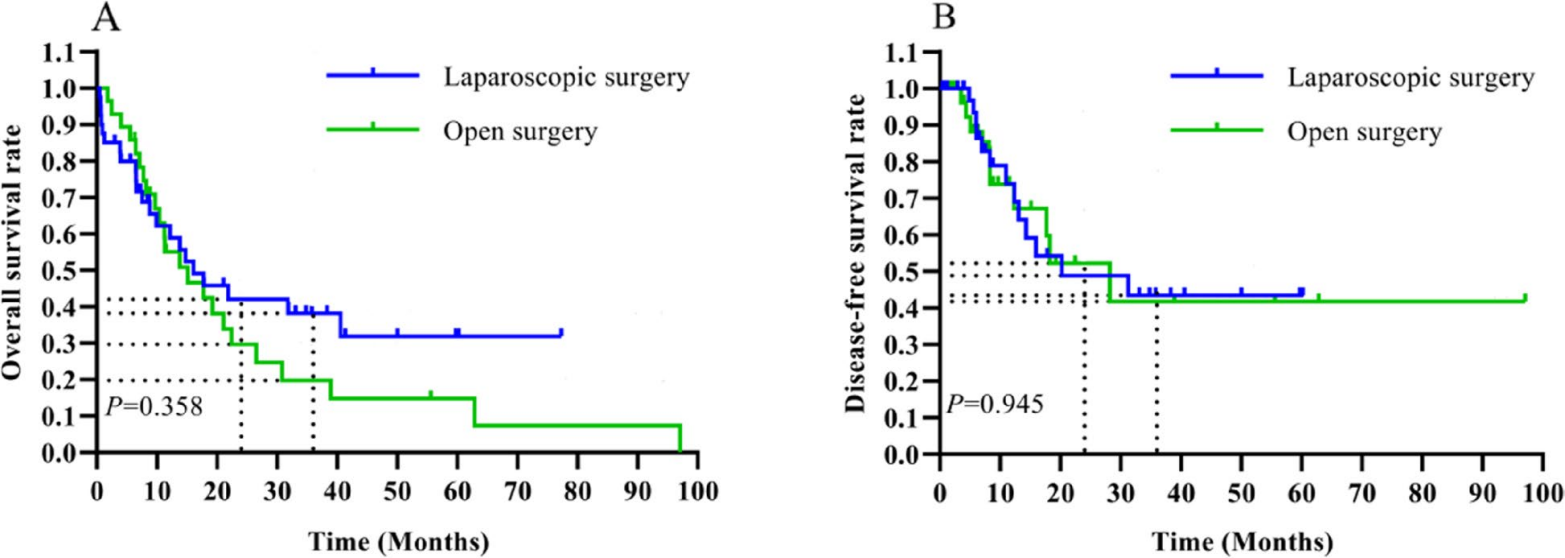
**A** Survival curve analysis of the overall survival time between the LS and OS groups; the difference was not statistically significant (*P* = 0.358). **B** Survival curve analysis of the DFS time between the LS and OS groups; the difference was not statistically significant (*P* = 0.945)

These findings, as detailed in Table 3 and illustrated in Fig. 6, contribute to our understanding of the long-term impact of surgical technique on patient outcomes following HCCA resection.

**Table 3:** Long-term prognosis of the LS and OS groups

Variable	LS group (*n* = 40)	OS group (*n* = 28)	*p*-value
Postoperative follow-up, months	9.37 (4.40–32.79)	11.42 (7.25–22.45)	0.478
Postoperative adjuvant therapy, *n* (%)	18 (45.0)	9 (32.14)	0.323
Chemotherapy	7 (17.5)	8 (28.57)	0.144
Chemotherapy + microwave ablation	2 (5.0)	0	
Chemotherapy + TACE	2 (5.0)	0	
Chemotherapy + targeted therapy	1 (2.5)	0	
Chemotherapy + immunotherapy	4 (10.0)	0	
Radiotherapy	1 (2.5)	0	
TACE	0	1 (3.57)	
Immunotherapy	1 (2.5)	0	
Total disease recurrence, *n* (%)	14 (35.0)	10 (35.71)	0.783
Way of recurrence, *n* (%)			
Locoregional relapse	9 (22.5)	7 (25.0)	> 0.999
Distant metastases	5 (12.5)	3 (10.71)	
Total death, *n* (%)	22 (55.0)	23 (82.14)	0.036
Causes of death, *n* (%)			
Other	13 (50.09)	13 (56.52)	> 0.999
Cancer progression	9 (40.91)	10 (43.48)	

*OS*, open surgery; *LS*, laparoscopic surgery; *TACE*, transhepatic arterial chemotherapeutic embolism. “*”Indicates a statistically significant difference (*P* < 0.05)

### Prognostic factor analysis for overall survival and DFS

In analyzing factors affecting survival outcomes, our univariate regression indicated radical resection and higher pathological differentiation as beneficial to extending overall survival. Moreover, preoperative biliary drainage and lymph node metastasis were significantly associated with overall survival, suggesting their importance in prognostication (see Appendix Table A1).

Our multivariate regression analysis revealed that patients younger than 65 years, those receiving radical resection, and those administered postoperative adjuvant therapy had improved overall survival rates. Conversely, preoperative biliary drainage emerged as a negative prognostic factor for survival (Table 4). For DFS, similar multivariate analysis identified perineuronal invasion as an independent adverse factor, highlighting its significance in recurrence risk assessment (Table 5).

**Table 4:** Cox multivariate regression analysis of factors affecting overall survival in HCCA

Parameter	HR	95% *CI*	*p*-value
Gender, female vs. male	0.940	0.460–1.910	0.867
Age, years (< 65 vs. ≥ 65)	0.380	0.150–0.940	0.036*
ECOG-PS, 0~1 vs. 2	1.920	0.710–5.210	0.202
Child-Pugh classification, B vs. C	1.610	0.690–3.740	0.268
Comorbidity, yes vs. no	0.950	0.360–2.490	0.912
Preoperative biliary drainage, yes vs. no	2.810	1.130–6.950	0.026*
CA-199, U/mL	1.000	1.000–1.000	0.895
Radical resection, yes vs. no	0.080	0.010–0.710	0.024*
Operation time, min	1.000	0.990–1.000	0.295
Hilar blockade, yes vs. no	2.170	0.910–5.180	0.081
Tumor size, < 3 cm vs. ≥ 3 cm	0.970	0.370–2.560	0.948
Surgical margin, R0/R1/R2	0.970	0.400–2.360	0.940
Bismuth type, I/II/IIIa/IIIb/IV	1.380	0.740–2.570	0.309
Pathological differentiation types, highly/moderately/poorly	0.770	0.410–1.440	0.409
Perineuronal invasion, yes vs. no	1.450	0.590–3.580	0.423
Lymph node metastasis, yes vs. no	2.140	0.990–4.630	0.053
Postoperative complications, yes vs. no	1.740	0.570–5.330	0.330
Postoperative adjuvant therapy, yes vs. no	0.380	0.150–0.960	0.040*
Treatment (LS vs. OS)	1.290	0.530–3.160	0.570

**Table 5:** Cox multivariate regression analysis of factors affecting postoperative recurrence of HCCA

Parameter	HR	95% *CI*	*p*-value
Gender, male vs. female	0.920	0.340–2.530	0.878
Age, years (< 65 vs. ≥ 65)	0.970	0.310–3.000	0.953
Preoperative biliary drainage, yes vs. no	2.050	0.660–6.420	0.217
CA-199, U/mL	1.000	1.000–1.000	0.679
CEA, μg/L	1.020	0.990–1.050	0.132
Radical resection, yes vs. no	0.640	0.030–11.910	0.764
Operation time, min	1.000	0.990–1.000	0.762
Tumor size, < 3 cm vs. ≥ 3 cm	1.940	0.430–8.670	0.387
Surgical margin, R0/ R1/ R2	2.420	0.580–10.030	0.223
Bismuth type, I/II/IIIa/IIIb/IV	1.410	0.570–3.490	0.463
Pathological differentiation types, highly/moderately/poorly	0.620	0.240–1.600	0.325
Perineuronal invasion, yes vs. no	5.180	1.170–22.960	0.030*
Intravascular tumor thrombus, yes vs. no	2.060	0.650–6.560	0.221
Lymph node metastasis, yes vs. no	2.100	0.670–6.610	0.206
Postoperative complications, yes vs. no	2.280	0.48–10.900	0.303
Postoperative adjuvant therapy, yes vs. no	1.270	0.360–4.510	0.710
Treatment (LS vs. OS)	0.920	0.260–3.260	0.896

*HR*, hazard ratio; *CI*, confidence interval; *ECOG-PS*, Eastern Cooperative Oncology Group performance status; *CA-199*, cancer antigen 19-9; *CEA*, carcinoembryonic antigen; *R0*, negative margin; *R1*, microscopic positive margin; *R2*, macroscopic residual tumor resection; *LS*, laparoscopic surgery; *OS*, open surgery. “*”Indicates a statistically significant difference (*P* < 0.05)

These analyses underscore the complexity of factors influencing survival and recurrence in HCCA, paving the way for tailored postoperative strategies to optimize patient outcomes.

## Discussion

Surgical resection is often considered the only effective option for treating HCCA [13, 14]. In recent years, a relatively small number of surgeons with extensive experience in laparoscopic techniques began exploring the development of a laparoscopic approach for radical resection of HCCA, with the first report of total laparoscopic HCCA resection appearing in 2011 [15]. Our present retrospective study included 68 HCCA patients. To the best of our knowledge, this is one of the largest retrospective, single-center cohort studies comparing LS and OS for HCCA in the world. Our results indicate that LS and OS did not significantly differ in clinical efficacy. Specifically, we observed comparable rates of overall survival beyond 2-year and 3-year DFS between the two groups. However, the 2-year DFS rate was lower in the LS group compared to the OS group. Our analysis further revealed that factors such as age under 65 years, radical resection, and postoperative adjuvant therapy were associated with a reduced risk of death, while preoperative biliary drainage was identified as an independent factor increasing the risk of death. Additionally, perineuronal invasion emerged as an independent risk factor affecting DFS. These findings contribute to the evolving understanding of laparoscopic versus open approaches in the treatment of HCCA and underscore the importance of patient selection and postoperative management strategies.

Few previous studies have compared the prognosis of HCCA patients who have undergone these two different types of surgical resection. Our findings, mirroring those of Qin et al. [16] in their study on LS and OP for perihilar cholangiocarcinoma, also indicate comparable long-term prognoses and short-term outcomes for both approaches. However, our study uniquely observed a higher rate of short-term complications and 90-day mortality in the LS group, likely due to our specific patient cohort and our center’s initial experience with laparoscopic HCCA surgery. This highlights the critical role of institutional experience and patient selection in surgical outcomes. The study by Ma et al. [17] on laparoscopic pancreaticoduodenectomy underscores the potential of laparoscopic techniques in complex abdominal surgeries, reinforcing the applicability of these approaches to HCCA. The evolution of specialized laparoscopic methods, as demonstrated in their research, opens avenues for improved surgical outcomes and recovery in HCCA treatment. Berardi et al.’s [18] systematic review emphasizes the significance of surgeon expertise and patient selection in laparoscopic liver resections, especially in challenging cases like HCCA. This aligns with our observations, suggesting that while laparoscopic methods are promising, their application must be carefully considered in complex hepatic surgeries. Berardi et al.’s insights further contextualize our findings within the broader challenges and opportunities of laparoscopic liver surgery.

Patients with HCCA are prone to caudal leaf metastases [14, 19]. Therefore, caudate lobectomy has gradually emerged as the global consensus approach for radical resection of HCCA tumors, but failure of caudate lobectomy may result in inadequate radical resection [20, 21]. However, hepatectomy or caudate lobectomy in laparoscopic radical resection of HCCA tumors is technically difficult and thus requires a high degree of expertise. In this study, left or right hemihepatectomy plus caudate lobectomy was notably more common in the LS group than the OS group, suggesting that the laparoscopic technique is beneficial for radical resection of HCCA tumors (Table 6). Our study also found that the OS group experienced more gallbladder swelling than the LS group (Table 6), possibly due to compression by the high pressure of carbon dioxide gas in the abdomen in LS patients. Additionally, magnification of the field of vision by the laparoscopic equipment enables the surgeon to better define the HCCA Bismuth-Corlette type, which aids in the formulation of HCCA intraoperative surgical plans and postoperative treatment plans, thereby improving treatment efficacy.

**Table 6:** Comparison of intraoperative parameters between the LS and OS groups

Variable	LS group (*n* = 40)	OS group (*n* = 28)	*p*-value
Radical resection, *n* (%)	34 (85.0)	27 (96.4)	0.226
Hilar blockade, *n* (%)	18 (45.0)	7 (25.0)	0.127
Intraoperative blood loss, mL	350 (200.0–600.0)	550 (400.0–800.0)	0.062
Intraoperative blood transfusion, *n* (%)			
Plasma, mL	12 (30.0)	17 (60.7)	0.614
Red cells, U	15 (37.5)	16 (57.1)	
Operation time, min	469 (422.5–520.25)	440 (342.5–510.0)	0.154
Liver resection, *n* (%)	36 (90.0)	20 (71.4)	0.060
No	4 (10.0)	8 (28.6)	0.041*
Minor liver resection	3 (7.5)	5 (17.9)	
Major liver resection	33 (82.5)	15 (53.6)	
Left hemihepatectomy (+ segment I)	24 (60.0)	11 (39.3)	
Right hemihepatectomy (+ segment I)	9 (22.5)	3 (10.7)	
Segment I resection, *n* (%)	25 (62.5)	7 (25.0)	0.003*
Left and right hepatic angioplasty, *n* (%)	16 (40.0)	10 (35.7)	0.720
Roux-en-Y reconstruction, *n* (%)	30 (75.0)	23 (82.1)	0.563
Conversion, *n* (%)	9 (22.5)	-	-
Surgical margin, *n* (%)			
R0	32 (80.0)	22 (78.6)	0.554
R1	3 (7.50)	4 (14.3)	
R2	5 (12.5)	2 (7.1)	
Vascular invasion, *n* (%)	23 (57.5)	12 (42.86)	0.234
Vascular reconstruction, *n* (%)	3 (7.5)	2 (7.14)	> 0.999
Lymphadenectomy, *n* (%)	38 (95.0)	23 (82.14)	0.115
Hilar plastic surgery, *n* (%)	8 (20.0)	8 (28.57)	0.562
Choledochojejunostomy to place support tube, *n* (%)	5 (12.5)	4 (14.29)	> 0.999
Biliary T-tube drainage, *n* (%)	3 (7.50)	4 (14.29)	0.435
Tumor size (cm)	3.07 ± 1.53	3.05 ± 0.88	0.949
Liver condition, *n* (%)			
Hepatic atrophy (left or right)	3 (7.50)	2 (7.14)	> 0.999
Cholestasis	25 (62.5)	15 (53.57)	0.462
Cirrhosis	18 (45.0)	7 (25.0)	0.127
Gallbladder condition, *n* (%)			
Empty gallbladder	9 (22.5)	5 (17.86)	0.765
Gallbladder swelling	9 (22.5)	14 (50.0)	0.018*
Gallbladder invasion	4 (10.0)	1 (3.57)	0.642
Bismuth type, *n* (%)			
I	0	1 (3.6)	0.005*
II	1 (2.50)	8 (28.6)	
IIIa	2 (5.0)	3 (10.7)	
IIIb	10 (25.0)	5 (17.9)	
IV	27 (67.5)	11 (39.3)	

Notation: *LS*, laparoscopic surgery; *OS*, open surgery; *R0*, negative margin; *R1*, microscopic positive margin; *R2*, macroscopic residual tumor resection; hilar blockade, temporary occlusion of the hepatic hilum during surgery to control blood flow and minimize blood loss; left and right hepatic angioplasty, surgical repair or unblocking of the hepatic arteries to ensure adequate blood flow to the liver lobes; Roux-en-Y reconstruction, a type of surgery to reconstruct the biliary tract after resection where the small intestine is joined to form a Y-shaped connection; hilar plastic surgery, a procedure to modify or repair the hepatic hilum, which is the area of the liver where the bile ducts, blood vessels, and nerves enter and exit. This surgery is often performed to improve the flow of bile and blood following tumor resection or to reconstruct the area after injury or disease. “*”Indicates a statistically significant difference (*P* < 0.05)

However, in our study, the decision to omit caudate lobe resection in the OS group was influenced by several factors. These included tumor characteristics such as size and invasion depth, where smaller tumors or those not extending into the caudate lobe allowed for less extensive resection. Patient-specific anatomical considerations, particularly variations in hepatic and portal venous anatomy, also played a role. The technical complexity and associated increased risks of complications such as bleeding and bile leakage were significant concerns, especially in patients with compromised liver function or other comorbidities. Furthermore, surgical decision-making was influenced by the surgeon’s expertise and real-time intraoperative assessment. In cases where the cancer had metastasized beyond the liver, or adjuvant therapy was planned, a more conservative approach was deemed appropriate. Patient preferences, informed consent, and institutional guidelines also guided our surgical strategy.

Regarding the 90-day mortality rates observed in our study, particularly the 17.5% in the LS group and 7.14% in the OS group, we recognize that these figures are higher than typically expected. This higher mortality rate in the LS group can be attributed to a combination of factors: The complexity of cases in our cohort was significant, often involving patients with advanced stages of disease and severe comorbidities, such as liver cirrhosis, which inherently increased surgical risks. Additionally, our center’s initial phase of adopting laparoscopic techniques for HCCA meant that the surgical team was still on a learning curve during the study period, potentially contributing to higher mortality rates. We also note that some of the patients included in the LS group were part of the early exploratory phase of laparoscopic HCCA surgery, where experience with complex laparoscopic procedures was still being developed. Furthermore, the high rate of cirrhotic patients in our study could also be a contributing factor, as patients with cirrhosis have an increased risk of complications following surgery. These insights are essential in understanding the context of the 90-day mortality rates and underline the need for continuous improvement in surgical techniques and patient selection criteria in the management of HCCA.

With regard to the prognosis of HCCA resection, patients in the LS group generally exhibited a low rate of postoperative morbidity, less pain, more-rapid recovery, took food earlier, and had a shorter hospital stay compared with the OS group [22, 23]. However, the postoperative complication rate was slightly higher in the LS group than the OS group, and the rate of mortality within 90 days was also higher in the LS group than the OS group (Table 2), possibly due to the inclusion of patients in the early exploratory phase of laparoscopic HCCA surgery. Moreover, the 12.5% rate of R2 resections within the LS group signifies a critical area for improvement. This emphasizes the challenges in patient selection for minimally invasive procedures and the importance of precise preoperative assessment. We propose employing advanced imaging techniques for accurate tumor delineation, intraoperative ultrasound to aid in defining resection margins, and considering neoadjuvant therapies to reduce tumor size preoperatively, which may collectively help decrease the rate of R2 resections.

Previous studies have reported longer overall survival and DFS in HCCA patients who underwent OS compared with those who underwent LS [24, 25]. Regarding these survival outcomes of HCCA resection, we observed trends suggesting a more favorable survival in the LS group beyond 2 years and the 3-year DFS compared to the OS group, although the difference was not significant (Fig. 6 A, B) (*P* > 0.05). It is important to note that these findings are based on unadjusted data. We acknowledge that unadjusted survival data may not account for potential confounding factors such as patient demographics, tumor characteristics, and other clinical variables. Therefore, while these trends are encouraging and indicative of potential benefits associated with laparoscopic techniques, they should be interpreted with caution. We recognize the need for further studies employing adjusted analysis methods to provide a more comprehensive understanding of the survival outcomes associated with LS and OS in HCCA treatment.

While we observed certain trends in our study suggesting potential benefits of LS in HCCA treatment, it is important to emphasize that these observations were not statistically significant when compared to OS. At present, the evidence does not conclusively favor one approach over the other in terms of clinical efficacy. Future advancements in laparoscopic technologies and techniques, as well as accumulating experience at high-volume centers, may provide further insights. However, any assertions about the future superiority of LS must be substantiated by robust clinical data and comparative studies. Therefore, ongoing research and technological development are essential to fully understand the potential role of LS in HCCA treatment.

Several previous studies revealed that the prognosis of HCCA resection is affected by multiple factors, including age at time of surgery, preoperative biliary drainage, the presence or absence of radical resection, resection margin status, tumor markers, pathological type, lymph node metastasis, and postoperative adjuvant therapy [26–30]. The present study combined clinical practice parameters and Cox univariate and multivariate regression analysis to screen the effect of these variables. Using this approach, Cox multivariate regression analysis found that age (< 65 years), radical resection, and postoperative adjuvant therapy were independently associated with prolonged overall survival, whereas preoperative biliary drainage was an independent hazard factor negatively associated with overall survival (Table 4). Despite the relatively small sample size, our study has adequate power to detect significant differences in survival outcomes between the two groups according to the power analysis using PASS software. Nevertheless, we recognize the importance of larger-scale studies for further validation and comprehensive understanding of the factors affecting postoperative prognosis in HCCA treatment. Previous studies reported that 38.8–84.5% of HCCA tumors involve the peripheral nerves [31–35]. The overall survival of patients without peripheral nerve involvement is markedly longer than that of patients with peripheral nerve involvement [36–38]. However, in a study of 20 cases of Bismuth-Corlette type III and IV HCCA using whole histological large slides, Wang et al. found features of peripheral nerve invasion in all of the patients examined [39]. Our study identified perineuronal invasion as an independent hazard factor that negatively affected DFS but had no significant effect on overall survival (Tables 4 and 5).

Additionally, it is pertinent to consider the role of transarterial radioembolization (TARE) within the spectrum of HCCA management, especially for cases deemed unresectable. TARE has been shown to offer a safe and effective palliative option, with some studies indicating its utility in down-staging tumors to resectable status[40]. Although our study concentrates on surgical interventions for resectable HCCA, acknowledging the emerging applications of TARE is crucial, as it reflects the comprehensive treatment modalities available and may inform future multimodal strategies.

The clinical implications of our findings are significant for surgical practice. The equivalence in survival outcomes between laparoscopic and open surgical approaches for HCCA offers the surgical team the flexibility to choose the method best suited to the patient’s condition and the hospital’s resources. This choice, informed by our study, can lead to improved patient care strategies where the benefits of minimally invasive surgery, such as reduced pain and quicker recovery, are weighed against the surgeon’s proficiency and experience with the technique. Furthermore, the identification of prognostic factors in HCCA surgery within our study can assist clinicians in preoperative planning and decision-making to enhance outcomes and tailor postoperative care to individual patient needs. Recognizing the importance of these clinical implications is essential for the continuous improvement of patient management in HCCA.

Our study has several limitations that warrant consideration. Firstly, as a retrospective study, it is subject to inherent selection bias. Secondly, while the power analysis using PASS software indicated a reasonable level of statistical power with a power value of 0.76, the sample size of 40 patients in the LS group and 28 in the OS group is relatively small. This limitation highlights the need for future studies with larger sample sizes to further validate our findings and to enhance the generalizability of the results. Thirdly, some of the data, particularly regarding tumor recurrence and mortality, were collected through telephone follow-up. This approach could be susceptible to memory recall bias, potentially impacting the accuracy of the information. Fourthly, the extensive time span covered by the study contributed to some data being incomplete, which might have influenced the study outcomes. Additionally, in our study, we employed the Bismuth-Corlette classification system, which has been internationally recognized for the classification of hilar cholangiocarcinoma. The Bismuth-Corlette system is particularly valuable for planning surgical resection and biliary reconstruction. We acknowledge that the TNM (tumor, node, metastasis) staging system is also a critical tool for assessing cancer prognosis; however, our dataset did not include TNM staging data. Going forward, we aim to integrate TNM staging into our data collection protocols to enhance the robustness of our survival analyses. Lastly, the study was conducted at a single center, limiting the broader applicability of the findings. Therefore, additional high-quality, multicenter studies, preferably prospective and controlled, are essential to confirm our results and to establish more definitive conclusions about the comparative effectiveness of laparoscopic and open surgeries for HCCA.

## Conclusions

Laparoscopic HCCA resection is associated with slightly higher overall survival and long-term DFS compared with OS. The age of HCCA patients (< 65 years old), preoperative biliary drainage, radical resection, and postoperative adjuvant treatment have an impact on their prognosis.

### Supplementary Information

**Additional file 1:** **Appendix: Table A1.** Cox univariate regression analysis of factors affecting overall survival in HCCA. **Table A2.** Cox univariate regression analysis of factors affecting postoperative recurrence of HCCA.

## Data Availability

No datasets were generated or analysed during the current study.

## Abbreviations

HCCA: Hilar cholangiocarcinoma
LS: Laparoscopic surgery
OS: Open surgery
DFS: Disease-free survival
ECOG: Eastern Collaborative Oncology Group
ASA: American Society of Anesthesiologists
CT: Computed tomography
MRI: Magnetic resonance imaging
MRCP: Magnetic resonance cholangiopancreatography
CBD: Common bile duct
CHA: Common hepatic artery
GDA: Gastroduodenal artery
LHA: Left hepatic artery
PV: Portal vein
LPV: Left portal vein
RHD: Right hepatic duct
BMI: Body mass index
PTCD: Percutaneous transhepatic cholangial drainage
ERCP: Endoscopic retrograde cholangiopancreatography
ICU: Intensive care unit
RMB: Renminbi
ARDS: Acute respiratory distress syndrome
DIC: Disseminated intravascular coagulation
TACE: Transhepatic arterial chemotherapeutic embolism
