# Supplementary material for: Survival analysis of laparoscopic surgery and open surgery for hilar cholangiocarcinoma: a retrospective cohort study
Source: World J Surg Oncol. 2024 Feb 19;22:58. doi: 10.1186/s12957-024-03327-3 (PMC10875844; doi:10.1186/s12957-024-03327-3)
Supplement: Supplementary file 1 — Additional file 1: Appendix: Table A1. Cox univariate regression analysis of factors affecting overall survival in HCCA. Table A2. Cox univariate regression analysis of factors affecting postoperative recurrence of HCCA. [file 12957_2024_3327_MOESM1_ESM.docx]

Table A1. Cox univariate regression analysis of factors affecting overall survival in HCCA

| Parameter | HR | 95% CI | *P* value |
| --- | --- | --- | --- |
| Gender, female vs. male | 1.117 | 0.592-2.109 | 0.733 |
| Age, years (<65 vs. ≥65) | 0.925 | 0.510-1.678 | 0.798 |
| BMI, kg/m2 | 0.952 | 0.854-1.063 | 0.382 |
| ASA score, I/II/III | 1.190 | 0.761-1.861 | 0.447 |
| ECOG-PS, 0~1 vs. 2 | 1.844 | 0.871-3.904 | 0.110 |
| Child-Pugh classification, B vs. C | 1.598 | 0.856-2.981 | 0.141 |
| Comorbidity, yes vs. no | 1.839 | 0.906-3.734 | 0.092 |
| Preoperative biliary drainage, yes vs. no | 2.546 | 1.345-4.819 | 0.004^*^ |
| ALT, U/L | 1.001 | 0.999-1.003 | 0.368 |
| AST, U/L | 1.000 | 0.997-1.003 | 0.896 |
| GGT, U/L | 1.000 | 0.999-1.000 | 0.464 |
| TBIL, μmol/L | 1.001 | 0.998-1.004 | 0.558 |
| DBIL, μmol/L | 1.002 | 0.997-1.006 | 0.453 |
| ALB, g/L | 1.001 | 0.996-1.006 | 0.827 |
| ALP, U/L | 1.000 | 0.999-1.001 | 0.679 |
| CA-199, U/mL | 1.000 | 1.000-1.000 | 0.049^*^ |
| CEA, μg/L | 1.004 | 0.988-1.020 | 0.637 |
| Radical resection, yes vs. no | 0.295 | 0.118-0.733 | 0.009^*^ |
| Operation time, min | 0.998 | 0.995-1.000 | 0.075 |
| Hilar blockade, yes vs. no | 1.766 | 0.958-3.255 | 0.068 |
| Intraoperative blood loss, mL | 1.000 | 0.999-1.000 | 0.287 |
| Tumor size, <3 cm vs. ≥3 cm | 1.371 | 0.690-2.721 | 0.368 |
| Liver resection, No/Minor/Major | 0.862 | 0.584-1.272 | 0.455 |
| Surgical margin, R0/ R1/ R2 | 2.026 | 1.309-3.137 | 0.002^*^ |
| Lymphadenectomy, yes vs. no | 1.302 | 0.511-3.318 | 0.580 |
| Bismuth type, I/II/IIIa/IIIb/IV | 1.334 | 0.853-2.085 | 0.207 |
| ICU admission, yes vs. no | 0.594 | 0.250-1.409 | 0.237 |
| Pathological differentiation types, Highly/Moderately/Poorly | 0.561 | 0.350-0.899 | 0.016^*^ |
| Perineuronal invasion, yes vs. no | 1.655 | 0.859-3.190 | 0.132 |
| intravascular tumor thrombus, yes vs. no | 0.816 | 0.399-1.669 | 0.577 |
| Lymph node metastasis, yes vs. no | 2.454 | 1.293-4.658 | 0.006^*^ |
| Postoperative complications, yes vs. no | 1.665 | 0.800-3.466 | 0.173 |
| Postoperative adjuvant therapy, yes vs. no | 0.753 | 0.409-1.385 | 0.361 |
| Tumor recurrence, yes vs. no | 1.377 | 0.756-2.508 | 0.296 |
| Treatment (LS vs. OS) | 1.320 | 0.729-2.393 | 0.360 |

Table A2. Cox univariate regression analysis of factors affecting postoperative recurrence of HCCA

| Parameter | HR | 95% CI | *P* value |
| --- | --- | --- | --- |
| Gender, male vs. female | 0.938 | 0.400-2.199 | 0.883 |
| Age, years (<65 vs. ≥65) | 0.902 | 0.404-2.014 | 0.801 |
| BMI, kg/m^2^ | 0.923 | 0.795-1.073 | 0.298 |
| ASA score, I/II/III | 0.872 | 0.472-1.609 | 0.660 |
| ECOG-PS, 0~1 vs. 2 | 1.415 | 0.477-4.201 | 0.532 |
| Child-Pugh classification, B vs. C | 1.100 | 0.456-2.658 | 0.832 |
| Comorbidity, yes vs. no | 1.250 | 0.517-3.021 | 0.621 |
| Preoperative biliary drainage, yes vs. no | 3.363 | 1.455-7.777 | 0.005^*^ |
| ALT, U/L | 1.002 | 0.999-1.004 | 0.247 |
| AST, U/L | 1.001 | 0.998-1.004 | 0.579 |
| GGT, U/L | 1.000 | 0.999-1.000 | 0.257 |
| TBIL, μmol/L | 1.000 | 0.996-1.003 | 0.834 |
| DBIL, μmol/L | 0.999 | 0.993-1.005 | 0.694 |
| ALB, g/L | 1.002 | 0.997-1.007 | 0.459 |
| ALP, U/L | 1.000 | 0.999-1.001 | 0.880 |
| CA-199, U/mL | 1.000 | 1.000-1.000 | 0.087 |
| CEA, μg/L | 1.017 | 0.998-1.037 | 0.084 |
| Radical resection, yes vs. no | 0.599 | 0.136-2.634 | 0.498 |
| Operation time, min | 1.000 | 0.996-1.004 | 0.998 |
| Hilar blockade, yes vs. no | 1.515 | 0.662-3.469 | 0.326 |
| Intraoperative blood loss, mL | 1.000 | 0.999-1.001 | 0.617 |
| Tumor size, <3 cm vs. ≥3 cm | 1.989 | 0.742-5.332 | 0.172 |
| Liver resection, No/ Minor/ Major | 0.988 | 0.576-1.696 | 0.965 |
| Surgical margin, R0/ R1/ R2 | 1.784 | 0.941-3.382 | 0.076 |
| Lymphadenectomy, yes vs. no | 1.321 | 0.392-4.444 | 0.653 |
| Bismuth type, I/II/IIIa/IIIb/IV | 1.489 | 0.816-2.716 | 0.194 |
| ICU admission, yes vs. no | 1.099 | 0.410-2.946 | 0.852 |
| Pathological differentiation types,  Highly/Moderately/Poorly | 0.416 | 0.218-0.792 | 0.008^*^ |
| Perineuronal invasion, yes vs. no | 2.590 | 1.126-5.959 | 0.025^*^ |
| intravascular tumor thrombus, yes vs. no | 1.918 | 0.837-4.394 | 0.124 |
| Lymph node metastasis, yes vs. no | 2.476 | 1.041-5.887 | 0.040^*^ |
| Postoperative complications, yes vs. no | 3.458 | 1.029-11.621 | 0.045^*^ |
| Postoperative adjuvant therapy, yes vs. no | 1.670 | 0.741-3.765 | 0.216 |
| Treatment (LS vs. OS) | 0.955 | 0.423-2.155 | 0.912 |

BMI, body mass index; ASA, American Society of Anesthesiologists; ECOG-PS, Eastern Cooperative Oncology Group performance status; ALT, alanine aminotransferase; AST, aspartate aminotransferase; GGT, gamma-glutamyl transpeptidase; TBIL, total bilirubin; DBIL, direct bilirubin; ALB albumin; ALP, alkaline phosphatase; CA-199, cancer antigen 19-9; CEA, carcinoembryonic antigen; R0, negative margin; R1, microscopic positive margin; R2, macroscopic residual tumor resection; ICU, intensive care unit; LS, laparoscopic surgery; OS, open surgery. “*”Indicates a statistically significant difference (*P*<0.05).
